# RP-HPLC Estimation of Ramipril and Telmisartan in Tablets

**DOI:** 10.4103/0250-474X.54283

**Published:** 2009

**Authors:** V. P. Kurade, M. G. Pai, R. Gude

**Affiliations:** Department of Pharmaceutical Analysis, Goa College of Pharmacy, Panaji-403 001, India

**Keywords:** Simultaneous estimation, RP-HPLC, ramipril, telmisartan, validation

## Abstract

A rapid high performance liquid chromatographic method has been developed and validated for the estimation of ramipril and telmisartan simultaneously in combined dosage form. A Genesis C18 column having dimensions of 4.6×250 mm and particle size of 5 μm in isocratic mode, with mobile phase containing a mixture of 0.01 M potassium dihydrogen phosphate buffer (adjusted to pH 3.4 using orthophosphoric acid): methanol:acetonitrile (15:15:70 v/v/v) was used. The mobile phase was pumped at a flow rate of 1.0 ml/min and the eluents were monitored at 210 nm. The selected chromatographic conditions were found to effectively separate ramipril (R_t_: 3.68 min) and telmisartan (R_t_: 4.98 min) having a resolution of 3.84. The method was validated in terms of linearity, accuracy, precision, specificity, limit of detection and limit of quantitation. Linearity for ramipril and telmisartan were found in the range of 3.5-6.5 μg/ml and 28.0-52.0 μg/ml, respectively. The percentage recoveries for ramipril and telmisartan ranged from 99.09-101.64% and 99.45-100.99%, respectively. The limit of detection and the limit of quantitation for ramipril was found to be 0.5 μg/ml and 1.5 μg/ml respectively and for telmisartan was found to be 1.5 μg/ml and 3.0 μg/ml, respectively. The method was found to be robust and can be successfully used to determine the drug content of marketed formulations.

Telmisartan is a new angiotensin II receptor antagonist for the treatment of essential hypertension usually given in combination with ramipril. Telmisartan (TEL) is chemically 4'-((1,4'-dimethyl-2'-propyl(2,6'-bi-1H-benzimidazol)-1'-yl)methyl)-(1,1'-biphenyl)-2-carboxylic acid. Ramipril (RAM) is chemically (1S,5S,7S)-8-((2S)-2-(((1S)-1-ethoxycarbonyl-3-phenyl-propyl)amino)propanoyl)-8-azabicyclo(3.3.0)octane-7-carboxylic acid. RAM is a highly lipophilic, long acting ACE inhibitor.

Literature survey revealed that telmisartan is not yet official in any of the pharmacopoeia. RAM is official in USP and BP where HPLC and potentiometric titration is the official method of analysis[1,2]. There are numerous methods reported for estimation of these drugs alone as well as in combination with other drugs in pharmaceutical dosage forms[3–8] and/or in biological fluids. However, no method has been reported so far for the estimation of these two drugs simultaneously in combined dosage forms. Hence, in the present study, a new reversed-phase high performance liquid chromatography method was developed and validated for the simultaneous estimation of RAM and TEL in tablets.

The liquid chromatographic system consisted of the following components: Knauer, Advanced Scientific Instruments containing Smartline Pump 1000, PDA detector and Rheodyne injector with 20 μl fixed loop. Chromatographic analysis was performed using Eurochrome software on a Genesis C18 column with 250×4.6 mm i.d. and 5 μm particle size. Analytically pure RAM and TEL were obtained as gift samples from M/s Cipla Ltd., Verna, Goa, India). Acetonitrile, methanol, water (E. Merck, Mumbai, India) were of HPLC grade, while orthophosphoric acid and potassium dihydrogenphosphate (KH_2_PO_4_) (S. D. Fine Chemicals, Mumbai, India) were of analytical grade used for the preparation of mobile phase. Tablet formulation containing labeled amount of 5 mg RAM and 40 mg TEL was procured from the local Pharmacy.

Potassium dihydrogenphosphate buffer (0.01 M) was prepared in water and pH was adjusted to 3.4 by using ortho phosphoric acid solution. The mobile phase components, buffer (pH 3.4): methanol:acetonitrile (15:15:70 v/v/v) were then mixed and finally filtered through a nylon membrane filters of 0.45 μ and sonicated. Around 50 mg of RAM and 50 mg of TEL were accurately weighed and transferred to a standard 100 ml and 50 ml volumetric flasks, respectively. To this 30 ml of methanol was added, shaken for 20 min and sonicated to dissolve the solids. After complete dissolution of the drugs, volume was made up to the mark with methanol to give stock solutions of 500 μg/ml of RAM and 1000 μg/ml of TEL separately.

A reverse phase C18 column equilibrated with the optimum composition of the mobile phase containing 0.01 M potassium dihydrogen phosphate buffer (adjusted to pH 3.4 with orthophosphoric acid):methanol:acetonitrile (15:15:70 v/v/v) was used to resolve the peaks of RAM and TEL. The mobile phase flow rate was maintained at 1 ml/min and effluents were monitored at 210 nm. The sample was injected using a 20 μl fixed loop, and the total run time was 10 min.

Appropriate aliquots of standard stock solutions of RAM and TEL were diluted with acetonitrile to obtain final concentrations in the range of 3.5-6.5 μg/ml of RAM and 28.0-52.0 μg/ml of TEL. The solutions were injected in triplicates for each concentration using a 20 μl fixed loop system and chromatograms were recorded. Calibration curves were constructed by plotting average content of the drug versus respective concentrations and regression equations were computed for RAM and TEL. The plots of average content Vs respective concentration of RAM and TEL were found to be linear in the range of 3.5-6.5 μg/ml and 28.0-52.0 μg/ml with coefficient of correlation (r^2^) 0.9963 and 0.9957 for RAM and TEL, respectively.

Ten tablets were weighed and finely powdered. Tablet powder equivalent to 5 mg of RAM and 40 mg of TEL was accurately weighed and transferred to a 100 ml volumetric flask. To this was added about 50 ml of methanol and flask was sonicated for 15 min. The flask was shaken, and the volume was made up to the mark with methanol. The above solution was then filtered through 0.45 μ Whatman filter paper. One millilitre of the above filtrate was further diluted up to 10 ml with acetonitrile to obtain final concentrations of 5 μg/ml and 40 μg/ml of RAM and TEL, respectively. Sample solution was then filtered using sample filtration assembly through nylon membrane filter of 0.45 μ.

The solution was injected under above chromatographic conditions and peak areas were measured. The quantification was carried out by keeping these values to the straight line equation of calibration curve. The method was validated for accuracy, precision, specificity, detection limit, quantitation limit and robustness as per ‘ICH’ guidelines[9].

Preanalyzed sample solution was spiked with ramipril and telmisartan in the same proportion as that present in the tablet formulation. Spiking was done at three different concentrations 70%, 100%, and 130% of the label claim. Accuracy of the method was studied by calculating the recovery of the spiked samples. The average recoveries are 99.09 to 101.64 and 99.45 to 100.99 for RAM and TEL, respectively.

Repeatability of the method was validated by performing six replicate assays of the homogeneous sample. Results were calculated in terms of %RSD of the content of RAM and TEL.

Method was also validated for intermediate precision by comparing the performance of the method on different day by different chemist. Six replicate assays of homogeneous sample were performed using the same procedure and chromatographic conditions, % RSD of the contents of RAM and TEL were calculated. This results and repeatability (performed on previous day, by different chemist) results were compared.

The specificity of the RP-HPLC method was determined by the complete separation of RAM and TEL as shown in (fig. 1) with parameters like retention time (t_R_), resolution (R_s_) and tailing factor (T). The peaks obtained for RAM and TEL were sharp and have clear baseline separation. Sample matrix did not show any interference with the analyte peaks. Retention times for RAM and TEL were 3.68 and 4.98 min, respectively. Different standard solutions were prepared in the concentration range of 0.1-2 μg/ml for RAM and 0.5-5 μg/ml for TEL and the limit of detection (LOD) and quantitation (LOQ) for ramipril and telmisartan was determined. Robustness of the method was studied by changing the flow rate of the mobile phase by ± 2% and also by observing the stability of the drugs for 24 h at room temperature in the dilution solvent.

**Fig 1:**
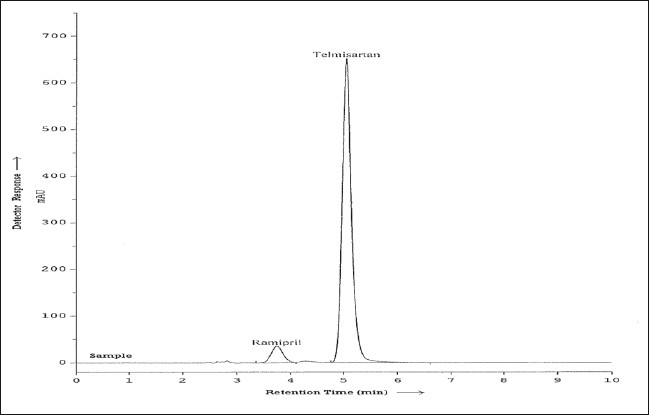
Typical sample chromatogram of RAM and TEL. Chromatogram of sample showing well resolved peaks of ramipril (RAM) at R_t_-3.68 min and telmisartan (TEL) with R_t_ of 4.98 min.

Optimization of mobile phase was performed based on resolution, tailing factor and peak area obtained for both ramipril and telmisartan. The mobile phase, 0.01 M potassium dihydrogen phosphate buffer (adjusted to pH 3.4 using orthophosphoric acid):methanol:acetonitrile (15:15:70 v/v/v) was found to be satisfactory and gave two symmetric and well-resolved peaks for RAM and TEL. The resolution between RAM and TEL was found to be 3.84, which indicates good separation of both the compounds. The retention time for ramipril and telmisartan were 3.68 min and 4.98 minute, respectively (fig. 1). The asymmetry factors for RAM and TEL were 1.01 and 1.19, respectively. The 3D dimension spectra of both RAM and TEL showed that both the drugs absorb appreciably at 210 nm so, 210 nm was selected as the detection wavelength in liquid chromatography.

The calibration curve for RAM was obtained by plotting the peak area of RAM versus the respective concentration of RAM over the range of 3.5-6.5 μg/ml, and it was found to be linear with r^2^ = 0.9963. Similarly, the calibration curve for TEL was obtained over the range of 28.0-52.0 μg/ml and was found to be linear with r^2^ = 0.9957. The detection limit for RAM and TEL were 0.5 μg/ml and 1.5 μg/ml, respectively. The quantitation limit for RAM and TEL were 1.5 μg/ml and 3.0 μg/ml, respectively, which suggest that a nanogram quantity of both the compounds can be estimated accurately. The validation parameters are summarized in (Table 1).

**TABLE 1:** SUMMARY OF VALIDATION PARAMETERS

Parameters	RAM	TEL
Detection limit (μg/ ml)	0.5	1.5
Quantitation limit (μg/ ml)	1.5	3.0
Accuracy (%)	99.09 - 101.64	99.45 - 100.99
Precision (RSD^a^, %)		
Repeatability (n=6)	0.917	0.981
Intermediate Precision (n=6)	0.754	0.827
Linearity (μg/ml)	3.5 - 6.5	28.0 - 52.0

a RSD indicates relative standard deviation; RAM is ramipril and TEL is telmisartan

The recoveries of ramipril and telmisartan were found to be in the range of 99.09-101.64% and 99.45-100.99 %, respectively. The system suitability test parameters are shown in (Table 2). The liquid chromatographic method was applied to the determination of RAM and TEL in their combined dosage forms (tablet formulation) and the average assay values were 98.94% and 99.84% of the labeled claim of RAM and TEL, respectively.

**TABLE 2:** SYSTEM SUITABILITY TEST PARAMETERS FOR RAMIPRIL AND TELMISARTAN BY THE PROPOSED METHOD

System Suitability Parameters	RAM	TEL
Retention time (min)	3.68	4.98
Resolution factor	-	3.84
Theoretical plates	1442.3	4686.7
Tailing factor (asymmetric factor)	1.01	1.19
RSD of Area* (%)	1.31	0.45
RSD of Rt* (%)	0.23	0.31

* mean of six observations

In the present study the attempt has been undertaken to develop most simple, economical, sensitive and accurate analytical HPLC method for the simultaneous estimation of these drugs without their prior separation. The method gives good resolution between both the compounds with a short analysis time (< 10 min). The method was validated and found to be simple, sensitive, accurate and precise. Percentage of recovery shows that the method is free from interference of the excipients used in the formulation. Therefore, the proposed method can be used for routine analysis of ramipril and telmisartan in their combined dosage form.
